# Exploring the preparation of YbBa_2_Cu_3_O_7−y_ superconductor in flowing oxygen atmosphere

**DOI:** 10.1038/s41598-024-59829-3

**Published:** 2024-04-18

**Authors:** Yanan Wang, Zerong Zhang, Zhan Gao, Lei Wang, Qiuliang Wang

**Affiliations:** 1 School of Rare Earths, University of Science and Technology of China, Hefei, 230026 China; 2 Ganjiang Innovation Academy, Chinese Academy of Sciences, Ganzhou, 341000 China; 3 Institute of Electrical Engineering, Chinese Academy of Sciences, Beijing, 100190 China

**Keywords:** High temperature superconductors, Yb123, Phase purity, Texture, Oxygen atmosphere, Materials science, Ceramics

## Abstract

REBCO has been used extensively as coated conductors applied to superconducting magnets due to its exceptional superconducting properties. As a REBCO superconductor, YbBa_2_Cu_3_O_7−y_ (Yb123) has a low melting temperature, making it suitable for use as an intermediate medium connector while preparing the superconducting joint. However, there is still uncertainty about the formation mechanism of Yb123 and the synthesis of this superconductor has not been fully understood. Therefore, this study systematically investigated the phase transformation process of Yb123 during heat treatment in flowing oxygen. The results indicated that Yb123 sample with the highest phase purity could be obtained by annealing at 927 °C or 937 °C but not in between, respectively. Furthermore, a quantitative phase analysis revealed that the sample annealed at 937 °C had a phase purity greater than 80 wt%. Additionally, a strong c-axis texture was observed in the bulk Yb123 superconductor prepared at 937 °C. Meanwhile, the superconducting results revealed that the bulk sample’s T_c_ was 89.9 K, and its self-field critical current densities at 4.2 K and 77 K were 1.3 × 10^5^ A/cm^2^ and 5.0 × 10^3^ A/cm^2^, respectively. Based on the results mentioned above, the phase transformation process and formation mechanism of Yb123 in flowing oxygen were elaborated.

## Introduction

Since the high temperature superconductors (HTS) were discovered in 1986^1^, REBa_2_Cu_3_O_7−y_ (abbreviated as REBCO or RE123, in which RE stands for rare earth elements), Bi_2_Sr_2_CaCu_2_O_x_ (Bi-2212) and Bi_2_Sr_2_Ca_2_Cu_3_O_10_ (Bi-2223) have drawn much attention due to their high operating temperatures, which are above the boiling point of liquid nitrogen^2^. Additionally, due to their excellent superconducting properties, HTS are commonly used in electromagnetic applications, such as high magnetic field magnets, superconducting cables, undulators, bearings and superconducting magnets for energy storage (SMES) and so on^3–8^.

Among the HTS, REBCO has been emerged as a promising superconductor, especially used as coated conductors applied to insert coils for ultra-high field nuclear magnetic resonance (NMR) devices because of its high irreversible field (H_irr_) and a comparatively low preparation cost when compared to Bi-2212/Bi-2223 superconducting tapes/wires. However, despite advancements in coated conductor preparation technology, the maximum length of REBCO tapes in current research is still approximately 1 km^9,10^, which is far from enough for long length cables and persistent current magnets for NMR/MRI. As a result, it is essential to fabricate a joint between two coated conductors given the requirement for the closed-loop persistent current operation of the coil, and the joint resistivity should be below 10^–12^ Ω·cm^2^^11–14^. To date, there have been multiple reports about the successful preparation of superconducting joints between REBCO tapes^15–22^, and the methods could be generally divided into two major categories. The first category is to fabricate the joint by direct diffusion bonding. Park et al. firstly tried to prepare a superconducting joint between GdBCO-coated conductors via direct melting diffusion and oxygen annealing in 2014. Although the superconductivity of the joint was fully recovered after oxygen annealing by this method, the annealing time exceeded 350 h and it was too long to be well applied in practice. The second category is to prepare the joint with the aid of immediate media, which could be superconducting solders or a layer of superconducting thin film. Specially, Jin et al. developed a joining technique called crystalline joint by a melted bulk (CJMB), in which the HTS tapes were jointed through another superconducting bulk with a lower melting temperature. By optimizing the heat treatment schedule, the melting and regrowth of immediate bulk could be fulfilled, and thereby achieving the preparation of a superconducting joint. It appears that adopting a REBCO material with a low melting temperature as the immediate media was crucial to the CJMB method.

Yb123 is one of the REBCO materials, and its T_c_ was about 86–90 K^23,24^. According to previous reports^25,26^, the melting temperature of REBCO material was closely related to the ion radius of RE, and Yb123 possessed a relatively lower melting temperature because of the smaller ion radius of Yb. Specially, it was reported that the melting temperature of Yb123 in air is over 80 K lower than that of GdBCO, and such a temperature difference was beneficial for subsequent joint preparation. Therefore, Yb123 superconductor was deemed as an ideal immediate material for CJMB method. While a few researchers report successfully obtaining single-phase Yb123^27^, the synthesis of high-purity Yb123 remains generally challenging. Among all rare earth ions used in REBCO, Yb^3+^ has the second-smallest ion radius, making it less favorable to form an ordered superconducting phase^28^. In particular, Yb211 is frequently observed and is difficult to completely eliminate^29–31^.

Given that the low melting temperature characteristic of Yb123, there have already been some reports and studies on the synthesis of Yb123 superconductor^29,32–36^. For example, as early as 1990, monophasic Yb123 was prepared at around 927 °C by Scmasundaram et al. by using about 8% excess copper in the initial starting composition, and the superconducting transition temperature was determined to be 89 K. However, there was no detailed description of the phase transformation process in the article. After that, Dwawha et al. attempted to synthesize Yb123 at 900 °C in air, and their results showed that a large amount of impurity phase of Yb211 was detected, meanwhile, the T_c_ and self-field J_c_ of the obtained Yb123 were about 86 K and 770 A/cm^2^ (77 K). In addition, Zhang et al. attempted to synthesize the Yb123 powder at 920 °C under flowing O_2_. However, the formation mechanism of Yb123 was not fully clarified, moreover, a certain amount of Yb211 was observed in the final Yb123 phase and the ratio of the strongest diffraction peak intensity of Yb123 to Yb211 was about 8. Overall, unlike the typical REBCO superconductor such as YBCO, the annealing process or reaction mechanism of YbBCO during preparation has not been fully understood, which in turn makes it difficult to synthesize Yb123 with high phase purity in most cases. Therefore, more work still needs to be done.

Thus, based on above analyses, in this work, the preparation of Yb123 superconductor in flowing oxygen was systematically investigated based on the Yb_2_O_3_-BaCO_3_-CuO system, and the reaction mechanism during heat treatment was discussed in detail.

## Methods

### Sample preparation

**Figure 1:**
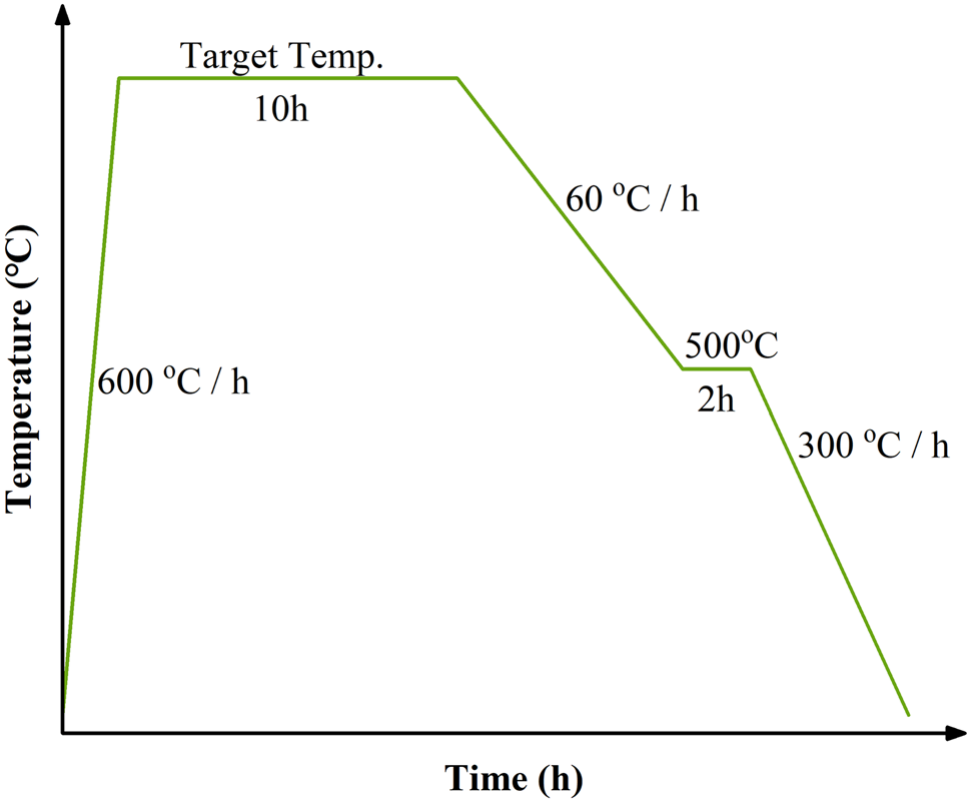
The heat treatment schedule for preparing Yb123 samples.

In the present work, Yb_2_O_3_ (99.99%), BaCO_3_ (99.99%) and CuO (99.5%) were purchased from Beijing Huawei Ruike Chemical Co., Ltd as starting materials and weighed according to the atomic ratio of Yb:Ba:Cu = 1:2:3. All the powders were firstly mixed by planetary ball milling, and then the Yb123 was synthesized by a solid-phase reaction method in the tube furnace equipped with high-precision temperature controller (SHIMAX, MAC 6A) and S-type thermocouple. Specifically, the raw sample was heated to the target temperature at a heating rate of 600 °C/h and held for 10 h. It was then cooled down to 500 °C at a rate of 60 °C/h and held for 2 h before cooling down naturally with furnace, as shown in Fig. 1. In this work, among all samples, the maximum annealing temperature was 950 °C and the lowest annealing temperature was 830 °C. Besides, the whole heat treatment process was conducted in flowing O_2_ atmosphere and repeated at least twice with the same heat treatment schedule. Moreover, note that no matter powder sample or bulk sample, a manual regrinding in an agate mortar for 15 min was performed between heat treatments.

In addition, the Yb123 bulk samples were prepared at 937 °C by the same sintering parameters as powder samples. The difference was that after the first sintering procedure, the obtained powders were then pressed uniaxially by a cylindrical mold with an inner diameter of 10 mm before second sintering. Moreover, in this work, the test bulk samples with specified dimensions were cut from the as-sintered bulks by diamond wire saw, and then successively wet-ground with SiC emery papers to 2000 grit, and polished with 0.5 μm diamond paste. At last, the samples were ultrasonically cleaned with alcohol before drying. And it was worth mentioning that the contact time between Yb123 and water must be short due to the hydrolysis of Yb123 while polishing^37,38^.

### Sample characterization

The phase constitutes of samples were identified by X-ray diffraction (XRD, Bruker D8 advance) with Cu Kα radiation (40 kV, 40 mA) at a scanning rate of 3°/min and a step size of 0.02°. The endothermic and exothermic peaks during the heating process were characterized by TG-DSC (NETZSCH STA 449F5). The temperature range was from 40 to 1000 °C with a heating rate of 10 °C/min and the whole process was carried out in flowing oxygen atmosphere. The surface morphologies were observed using field emission scanning electron microscope (FE-SEM, JEOL JSM-IT800) equipped with an energy dispersive spectroscopy (EDS, Oxford Instrument). The microstructures were further characterized by transmission electron microscope (TEM, FEI Talos F200X G2) operated at 200 kV with an EDS system. The sample for TEM observation was prepared by a dual-beam focused ion beam (FIB, Helios G4Ux) system. Specifically, a Pt layer was firstly deposited on the sample surface for protection, then milled to the desired thickness and polished by Ga ion beam.

The magnetic moment versus temperature (M-T) curves were measured from 10 to 110 K by applying a magnetic field of 1 mT along the c-axis direction employing superconducting quantum interference device (SQUID, Quantum design MPMS3). To estimate the field dependence of J_c_, magnetic hysteresis (M-H) loops were measured under a magnetic field up to 5 T at 4.2 K, 10 K, 20 K and 77 K, respectively. Moreover, in this work, J_c_ (A/cm^2^) was calculated based on the bulk samples utilizing the extended Bean’s critical state model^36^:

1 $$\text{Jc } = \frac{{20}\Delta {\text{M}}}{{\text{a}}\left({1}-\frac{\text{a}}{{\text{3b}}}\right) \, {\text{V}}},$$

where $\Delta$ M (emu) is the difference in the magnetic moment at the same magnetic field in the M-H loops, in addition, a and b (a ≤ b) are the dimensions (cm) perpendicular to the applied external magnetic field, and V is the volume of the specimen. In this work, the typical sizes of the test specimen were a = 1.7 mm, b = 1.7 mm and c = 2.0 mm, respectively.

## Results

### XRD analyses of as-prepared Yb123 samples

**Figure 2:**
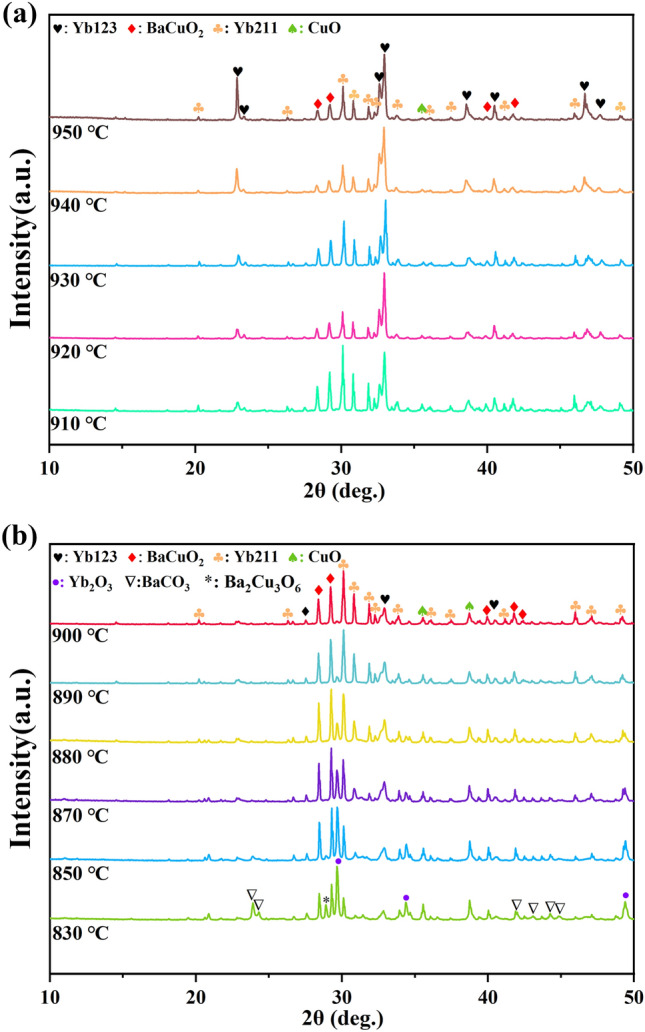
The XRD patterns of powder samples annealed at (**a**) 910 °C to 950 °C, and (**b**) 830 °C to 900 °C.

The XRD patterns of as-prepared Yb123 powder are presented in Fig. 2. It is seen that in the heat treatment temperature range of 830 °C to 950 °C, besides the raw materials, Yb123, Yb211 and BaCuO_2_ were mainly detected. When the temperature was below 910 °C, as shown in Fig. 2b, Yb123 was not the principal phase. In specific, at 830 °C, Yb_2_O_3_, CuO and BaCO_3_ were identified, and only a minor amount of reaction phase, such as Yb211 and BaCuO_2_, was recognized, indicating that the raw powders had not been fully reacted yet in the case. As the temperature increased to 850 °C, BaCO_3_ was fully reacted and the peak intensities of Yb211 and BaCuO_2_ got increased significantly. Afterwards, Yb211 was gradually converted into the principal phase as the temperature increased to 890 °C. By further increasing the annealing temperature, it was found that the peak intensity of Yb123 was greatly enhanced, and Yb123 was gradually transformed to the principal phase, as shown in Fig. 2a. Moreover, up until 950 °C, Yb123 was maintained as the principal phase. Additionally, CuO could be detected by XRD at the whole temperature range.

**Figure 3:**
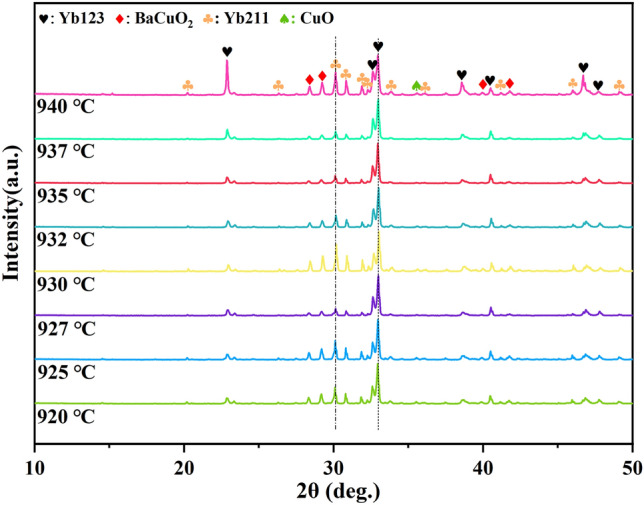
The XRD patterns of Yb123 powder samples annealed at 920 °C to 940 °C.

However, at closer observation, as shown in Fig. 2a, it could be found that the peak intensities of Yb211 and BaCuO_2_ underwent a transition from 920 to 940 °C although the principal phase was maintained as Yb123. Specifically, the peak intensities of Yb211 and BaCuO_2_ at 930 °C were higher than that at 920 °C and 940 °C. In other words, some unknown reactions were probably occurred within this temperature range. Therefore, further annealing treatment was carried out starting from 920 °C and increasing by 2–3 °C per time until 940 °C to verify the above transition, and the results are shown in Fig. 3. Unsurprisingly, Yb123 was the principal phase at each annealing temperature. Moreover, it can be seen that the diffraction peaks of Yb123 appear to shift slightly towards higher angles for the samples annealed at intermediate temperatures. In combination with the chemical formula of YbBa_2_Cu_3_O_7−x_, it was inferred that the shift of diffraction peaks was concerned with the oxygen content in Yb123^39^.

**Figure 4:**
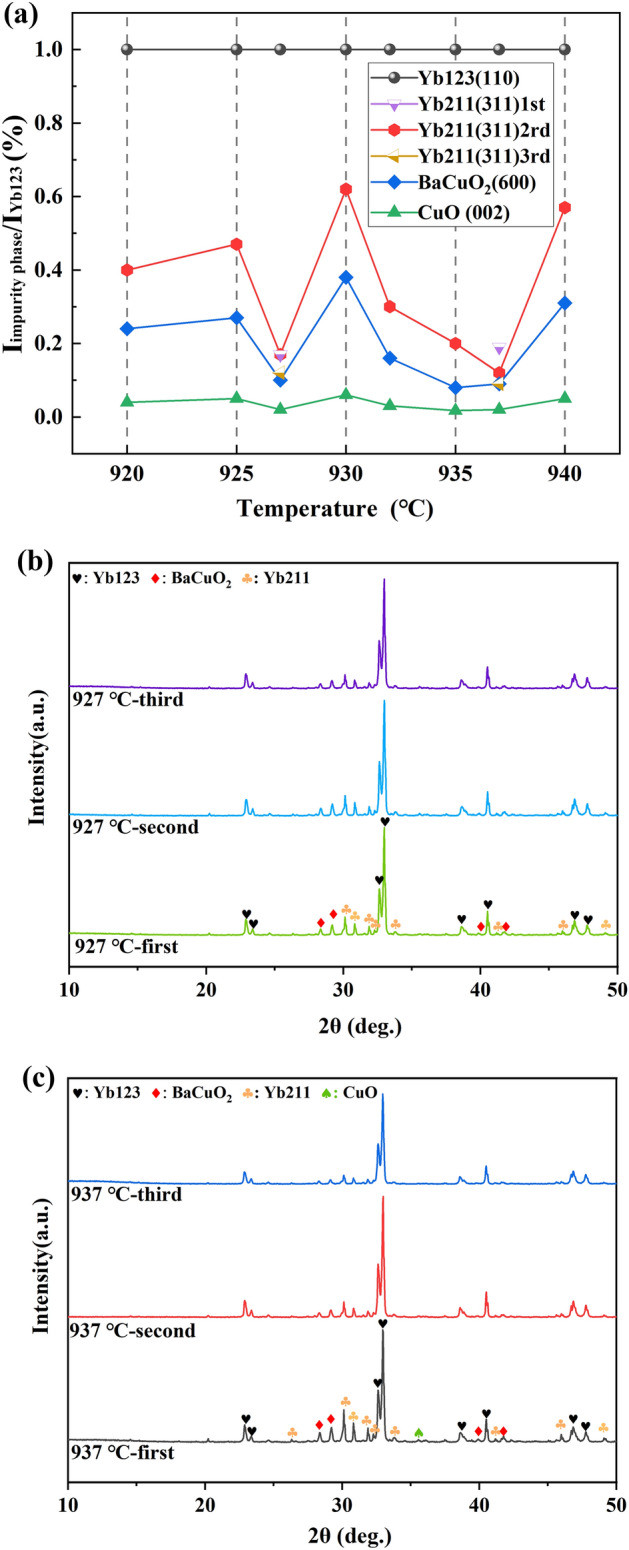
(**a**) The ratios of the peak intensity of impurity phases to Yb123 (110) at different temperatures, and the XRD results annealed at (**b**) 927 °C and (**c**) 937 °C for three times.

Furthermore, considering that it was not easy to tell the differences in the variation of peak intensities based solely on the XRD patterns, therefore, the variation of phase purity, characterized by peak intensity herein, was further analyzed based on the ratio of characteristic diffraction peak intensity of impurity phase (including Yb211, BaCuO_2_ and CuO) to Yb123. In specific, the strongest diffraction peak of Yb123, which is the (110) peak (2θ = 33.0°), was selected as a reference for normalization, thereby analyzing the changes of the peak intensities of Yb211 (311), BaCuO_2_ (600) and CuO (002), and the results are shown in Fig. 4a. Unexpectedly, two minimum points respectively located at 927 °C and 937 °C were observed, suggesting that annealing at 927 °C or 937 °C could result in a higher phase purity of Yb123. In other words, the decomposition and resynthesis of Yb123 was probably occurred in the temperature range of 925 °C to 940 °C, and such a phenomenon, to our knowledge, was first observed during sintering of Yb123 and other REBCO materials.

In addition, it should be note that the above XRD results were obtained after a second heat treatment. Therefore, based on above analyses, a third heat treatments was conducted at 927 °C and 937 °C with the hope of further improving the phase purity, and all the XRD patterns are presented in Fig. 4b,c. It was found that the phase purity of Yb123 was indeed slightly improved after a second heat treatment, however, the results of the third and the second heat treatments were basically the same. And the ratios of the peak intensity of Yb211 to Yb123 after the first and the third heat treatments were also depicted in Fig. 4a.

**Figure 5:**
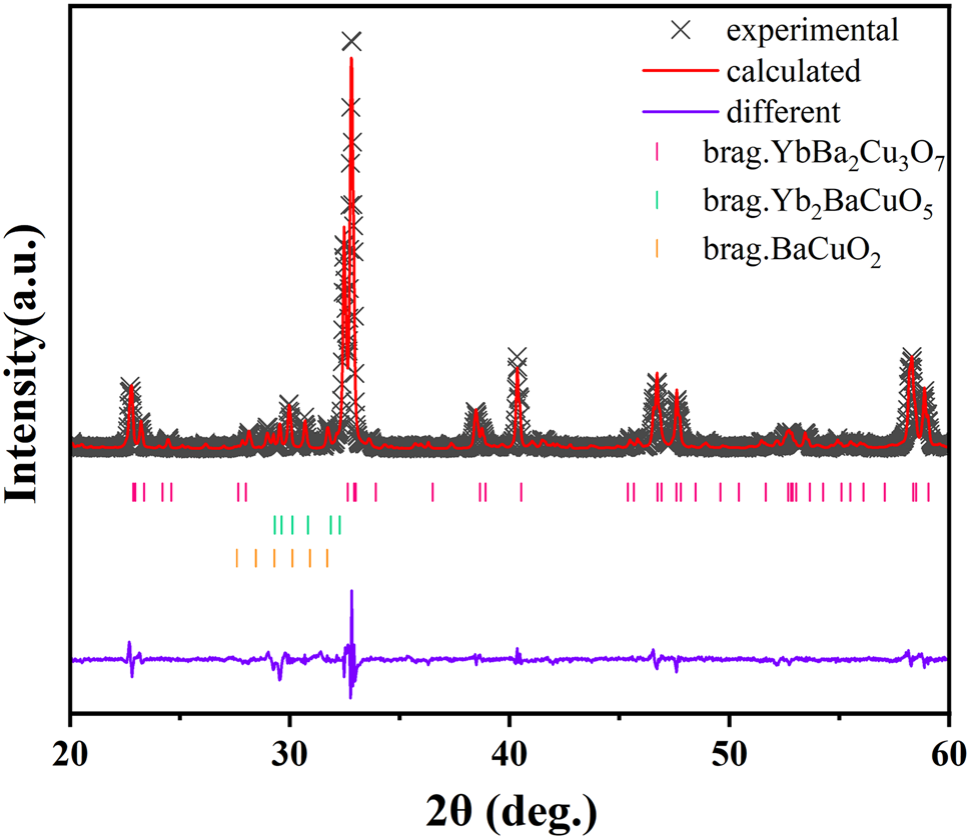
The fitting results of the XRD pattern for Yb123 powder sample sintered at 937 °C for three times by Rietveld refinement.

Furthermore, the quantitative phase analysis was conducted by Rietveld refinement based on the XRD pattern sintered at 937 °C for three times to determine the phase content of Yb123, as shown in Fig. 5 and Table 1^40^. The ratio of the peak intensity of Yb123 (110) to Yb211 (311) reaches 8.7, and the content of the target phase of Yb123 reaches 81.9 wt%, which is higher than that reported in most of literatures^28,30,41^.

**Table 1:** The phase contents and structural parameters of Yb123, Yb211 and BaCuO_2_.

Compound	Content (wt%)	Space group	a (Å)	b (Å)	c (Å)
YbBa_2_Cu_3_O_7_	81.90	Pmmm	3.78	3.85	11.59
Yb_2_BaCuO_5_	12.02	Pnma	11.99	5.58	7.01
BaCuO_2_	6.08	Im-3m	18.29	18.29	18.29

Additionally, according to above XRD results presented in Fig. 2a, preferential orientation was observed in the XRD patterns of the bulk samples annealed at higher temperatures. Specifically, for the samples annealed at 940 °C and 950 °C, the (003) peak (2θ = 23.0°) of Yb123 phase was significantly enhanced compared with other samples. Based on previous reports^35,42^, the generation of liquid phase was generally necessary prior to the growth of the texture for REBCO bulks, and the melting of powders was also observed in this experiment. Moreover, given that Yb123 with a high phase purity could be obtained^24^ after annealing at 927 °C and 937 °C, therefore, the annealing temperature for preparing Yb123 bulk samples was determined to be 937 °C in the hope of obtaining high-purity and c-axis oriented Yb123 bulks.

**Figure 6:**
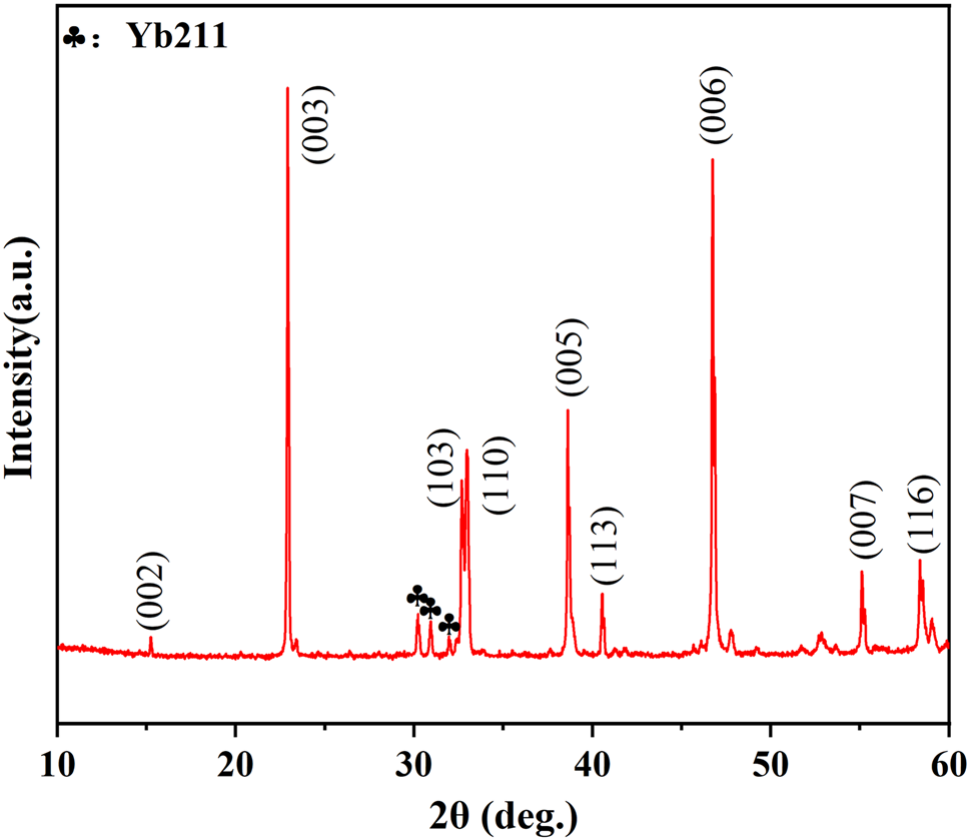
The XRD pattern of Yb123 bulk sample annealed at 937 °C in flowing oxygen.

The XRD pattern of the Yb123 bulk sample is shown in Fig. 6. As expected, c-axis texture can be clearly observed, and the c-axis direction of the bulk is the same as the cold pressing direction. The peak intensities of Yb123 (00Ɩ) peaks were significantly higher than other peaks. Meanwhile, only a minor amount of Yb211 phase was detected. Herein, it should be noted that texture had not been observed for the powder sample annealed at 937°C as shown above, indicating that in this work cold pressing was a primary step for the growth of texture in the preparation of bulk sample. This was because the act of pressing broke the mechanical symmetry of the precursor system and facilitated the formation of texture. On such a basis, cold pressing also improved the powder contact and facilitated the generation of liquid phase, ultimately promoting the growth of texture in bulk samples.

### Phase transformation during heat treatment

**Figure 7:**
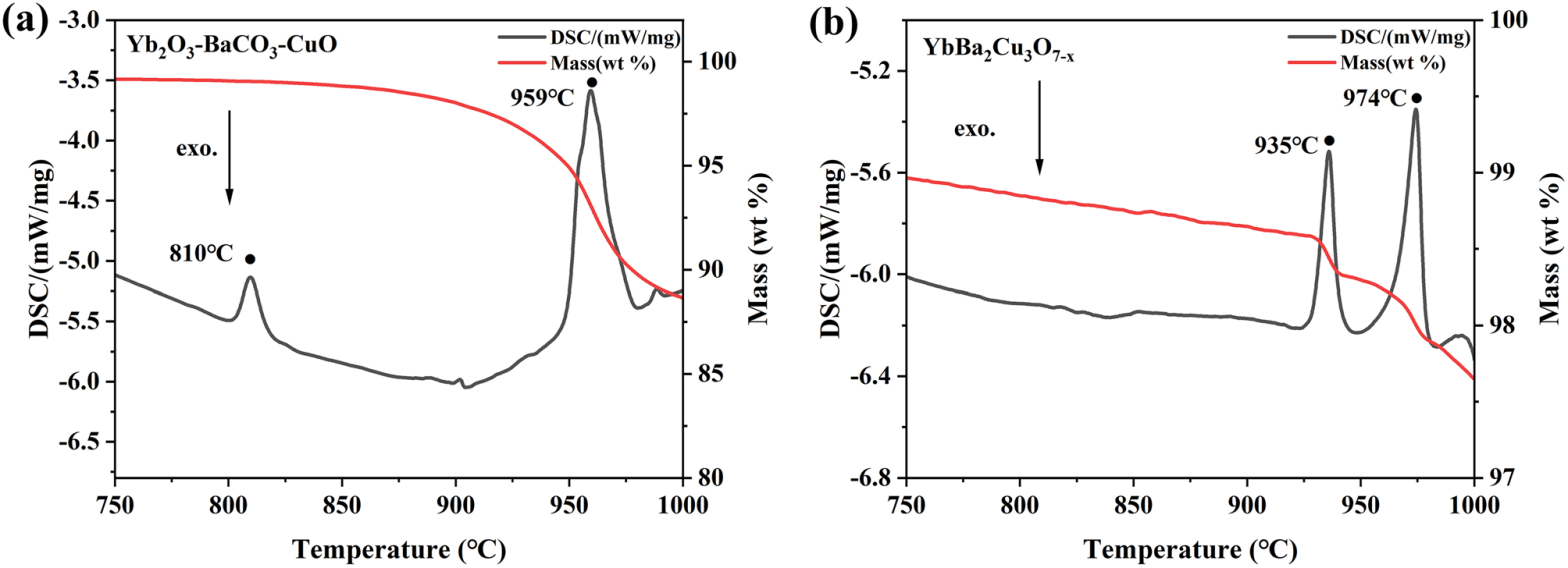
The TGA/DSC results of the samples tested in the form of (**a**) raw powders and (**b**) the as-prepared Yb123 annealed at 927 °C.

To figure out the phase transformation process during heat treatment, thermal analyses were carried out firstly based on the raw powders of Yb_2_O_3_–BaCO_3_–CuO system, and the DSC curve is presented in Fig. 7a. Apparently, two endothermic peaks were observed at 810 °C and 959 °C, respectively. According to previous reports^43–46^, BaCO_3_ is transformed from the orthorhombic α phase (*P*mcn) to the trigonal β phase (*R*3m) at 811 °C. Therefore, it could be deduced that the endothermic peak at 810 °C should be attributed to the phase transformation of BaCO_3_. Herein, it should be noted that although the phase content of BaCO_3_ was significantly reduced above 830 °C based on the XRD results in Fig. 2b, the endothermic peak concerned with the decomposition of BaCO_3_ had not been detected. The disappearance of BaCO_3_ was probably due to the reactions with other raw materials rather than decomposing into carbon dioxide directly, and the actual decomposition temperature reported in the literature was higher than 850 °C^45^. For the second endothermic peak at 959 °C, considering that at this short time scale, a large quantity of Yb123 is unlikely to form via solid-state reaction between milled powders, therefore, the large endothermic peak could not be originated from the decomposition reaction of Yb123. According to previous report^47^, Ba–Cu–O eutectic subsystem melts at 937℃ in 1 atm O_2_ at a heating rate of 1 °C/min, and the delay of endothermic peak could be observed due to a higher heating rate. Therefore, it was inferred that the endothermic peak at 959 °C in this work was probably concerned with the melting of the Ba–Cu–O eutectic subsystem according to Eq. (2):

2 $$ {\text{Ba-Cu-O}} + {\text{ CuO }} \leftrightarrow {\text{ L }}\left( {\text{liquid phase}} \right) \, + {\text{ O}}_{{2}} \uparrow . $$

In addition, according to the TGA curve in Fig. 7a, the mass loss starts to appear below 900 °C, and the Ba–Cu–O phase should be formed before the occurrence of Eq. (2), therefore, it was inferred that the reaction (3), see as follows, was also occurred prior to endothermic peak.

3 $$ {\text{BaCO}}_{{3}} + {\text{ CuO }} = {\text{ BaCuO}}_{{2}} + {\text{ CO}}_{{2}} \uparrow . $$

Furthermore, to figure out the decomposition behaviors of Yb123, DSC analysis was carried out using the as-prepared Yb123 powder annealed at 927 °C as the test sample, as shown in Fig. 7b. Two endothermic peaks are also observed at 935 °C and 974 °C, respectively. According to previous reports^48^, the first endothermic peak in Fig. 7b was concerned with the reaction between Yb123 and CuO impurity phase, as expressed by Eq. (4):

4 $$ a{\text{YbBa}}_{{2}} {\text{Cu}}_{{3}} {\text{O}}_{{{7} - {\text{y}}}} + b{\text{CuO }} \leftrightarrow c{\text{Yb}}_{{2}} {\text{BaCuO}}_{{5}} + d{\text{L }} + e{\text{O}}_{{2}} \uparrow . $$

Additionally, the second endothermic peak at 974 °C was induced by the melting of Yb123 as expressed by Eq. (5):

5 $$ {\text{YbBa}}_{{2}} {\text{Cu}}_{{3}} {\text{O}}_{{{7} - {\text{y}}}} \leftrightarrow a{\text{Yb}}_{{2}} {\text{BaCuO}}_{{5}} + b{\text{L }} + c{\text{O}}_{{2}} \uparrow . $$

**Figure 8:**
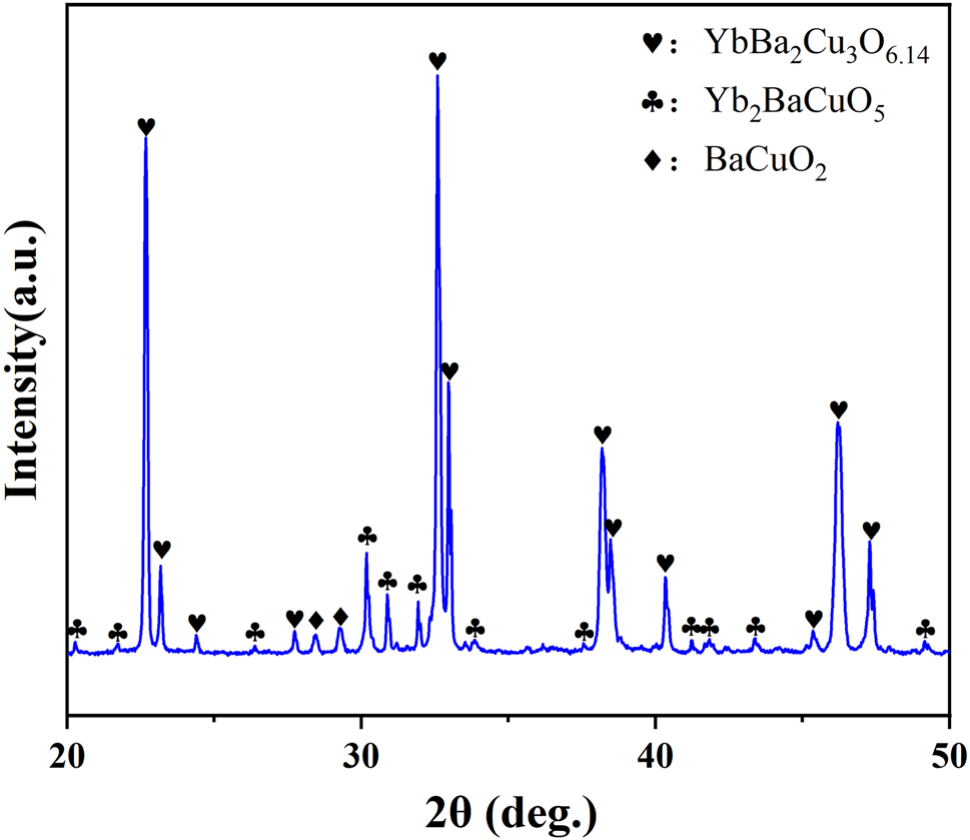
The XRD pattern of Yb123 bulk sample quenched from 937 °C.

Moreover, to further clarify the reactions occurred at this temperature range, the quenching experiment was conducted. In specific, the Yb123 pellet was immediately taken out of the tube furnace after holding at 937 °C for 10 h and quenched in liquid nitrogen, and the XRD result is shown in Fig. 8. It is seen that besides Yb123 and Yb211, BaCuO_2_ was also detected. Additionally, it was revealed that compared with the XRD result for the bulk sample annealed at 937 °C (as shown in Fig. 6), the c-axis texture got significantly weakened for the sample quenched from 937 °C (as shown in Fig. 8), which indicated that the cooling process in the furnace was crucial for the growth of texture. In addition, it should be noted that the diffraction peaks of Yb123 in Fig. 8 were matched with the phase of YbBa_2_Cu_3_O_6.14_ (PDF#823-2310), in which the oxygen content was below 6.5.

### Microstructure and composition analyses

As presented above, high phase purity and c-axis oriented Yb123 bulk sample could be obtained by annealing at 937 °C. Given that such results could probably provide a reference for the preparation of other REBCO bulks, therefore, further characterizations of the microstructure and composition were carried out on the as-prepared Yb123 bulk in this work.

**Figure 9:**
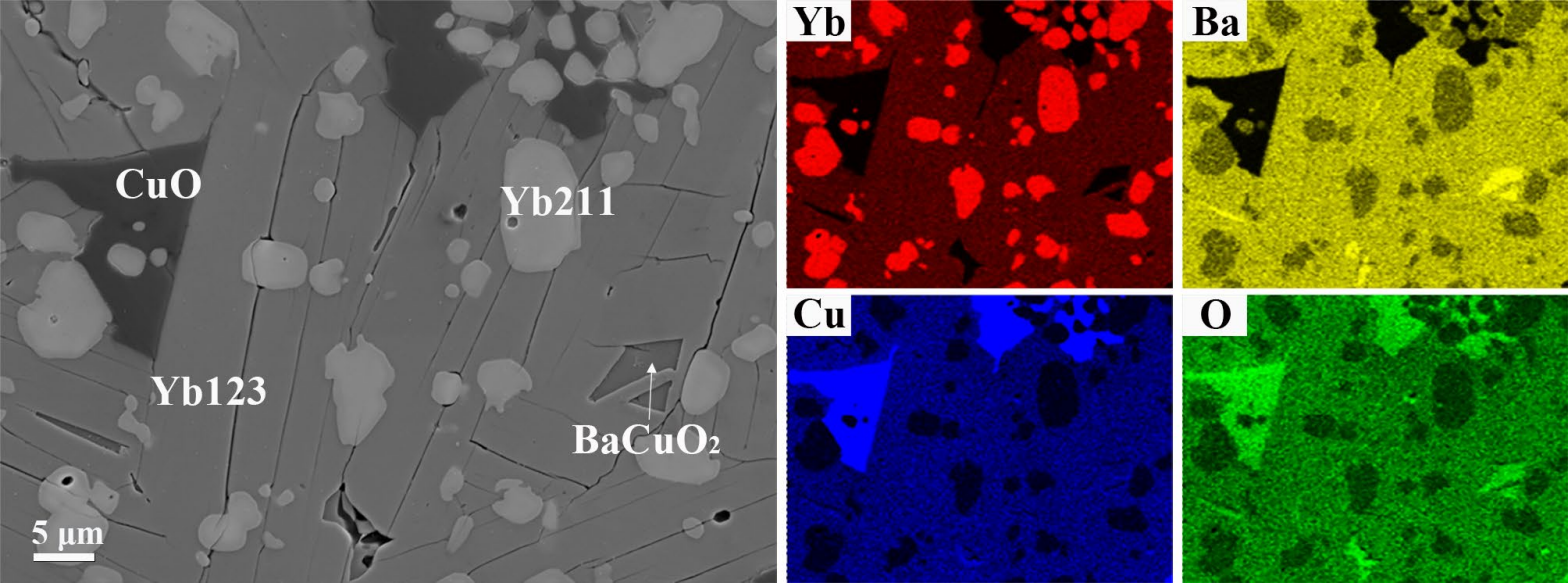
SEM image and the elemental distribution results of Yb123 bulk annealed at 937 °C.

The SEM results are presented in Fig. 9. According to the elemental distribution results, Yb211, CuO and a minor amount of BaCuO_2_ were identified in Yb123 matrix. Therefore, CuO and BaCuO_2_ were not detected by XRD in Fig. 6 mainly because their contents were too low and below the detection limit of XRD instruments. By analyzing the contours of various phases, it was found that the CuO was irregular. In contrast, the edges of the Yb211 phase were smooth and the average size is about several micrometers. In addition, the Yb211 phase was not well distributed, which was perhaps concerned with occurrence of liquid phase during annealing^49^. Moreover, the Yb123 phase exhibits slat-shape morphology, and the crevices between slats are clearly visible. Considering that this was a highly c-axis oriented sample, such a morphology was probably related to its growth of texture.

**Figure 10:**
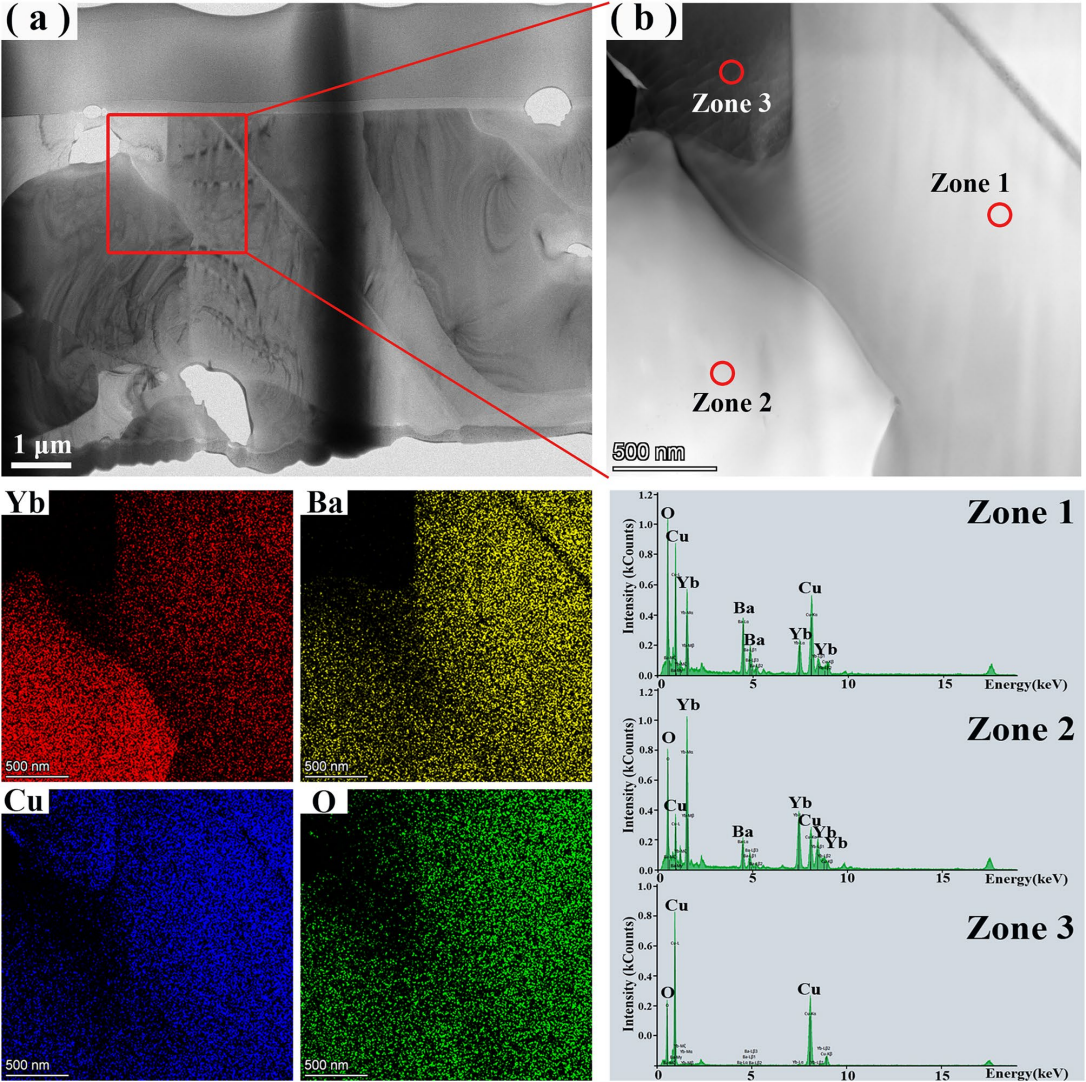
The TEM results of (**a**) bright field image, and (**b**) HAADF image for the Yb123 bulk annealed at 937 °C. The corresponding elemental distribution and point scanning results were also displayed.

In order to further characterize the microstructures of Yb123 bulk, TEM analyses were conducted, as shown in Fig. 10. In combination with the bright field image and the high angle annular dark field (HAADF) image, three different zones can be identified. Furthermore, based on the elemental distribution and point scanning results, it could be found that dark zone (namely Zone 3) in Fig. 10b corresponds to CuO. The white zone (namely Zone 2) and grey zone (namely Zone 1) are respectively corresponding to Yb211 and Yb123.

**Figure 11:**
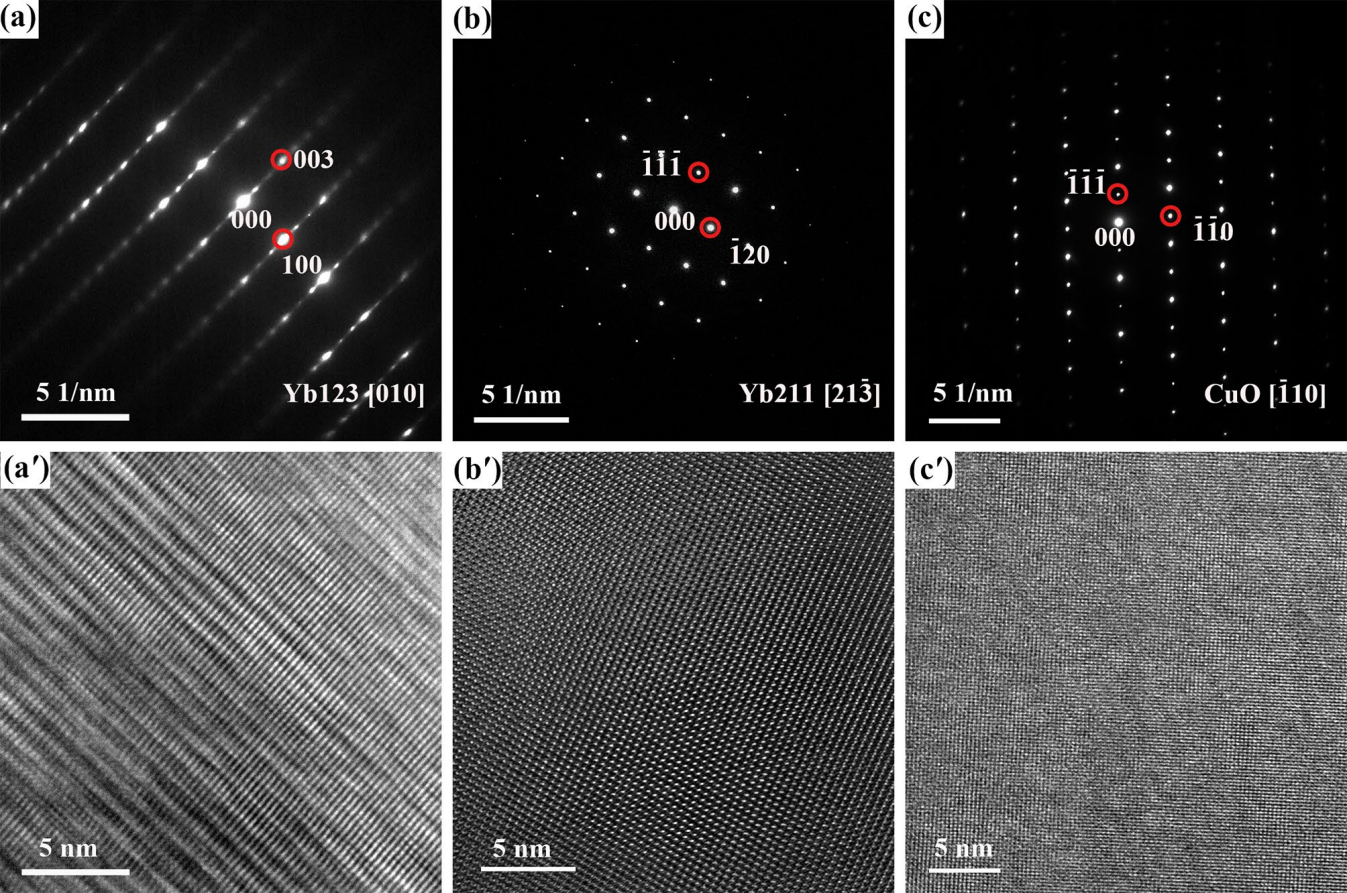
The SAED images of (**a**) Zone 1, (**b**) Zone 2 and (**c**) Zone 3, accordingly, (**a′**–**c′**) were corresponding HRTEM images.

Additionally, the selected electron diffraction (SAED) images and the high resolution TEM (HRTEM) images are presented in Fig. 11. As expected, the phases of Yb123, Yb211 and CuO were further verified. Moreover, it was worth mentioning that the (00Ɩ) texture of Yb123 bulk was also identified by the SAED result, as shown in Fig. 11a.

### Superconducting properties

**Figure 12:**
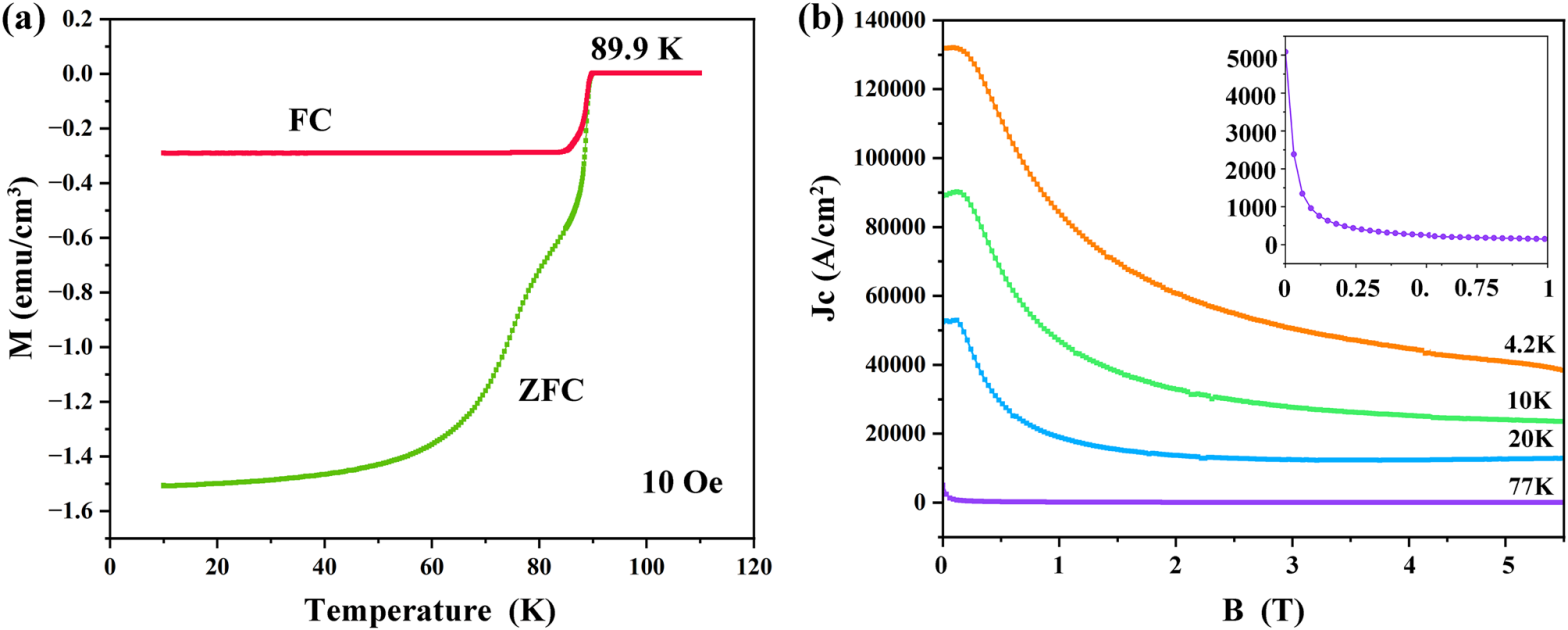
(**a**) Dependence curves of magnetization on temperature, and (**b**) the field dependence of the critical current density at 4.2 K, 10 K, 20 K and 77 K.

The superconducting properties of the Yb123 bulk sample annealed at 937 °C were measured and the results are shown in Fig. 12. The typical curves of temperature dependence of magnetization are demonstrated in Fig. 12a. It was revealed that the superconducting transition temperature (T_c,onset_) is about 89.9 K, which is higher than that reported in previous literatures^35,50^, and this was probably concerned with the purity of superconducting phase. In addition, a double transition was observed in M-T curve. Generally, such a double platform can be induced by the existence of two superconducting phases, the inhomogeneity of the sample and the influence of flux pinning effect. In this work, considering that the oxygen annealing time (2 h at 500 °C) was relatively short, the double transition might be the result of inhomogeneous oxygenation of the bulk Yb123^51^. The calculated critical current densities (J_c_) at different temperatures based on the M-H curves (not shown here) and Bean’s model are plotted in Fig. 12b^32^. The self-field J_c_ at 4.2 K exceeds 130 kA/cm^2^, and this value got decreased as the temperature increased. At 77 K, the self-field J_c_ is barely 5 kA/cm^2^, which was one order magnitude lower than the common single crystalline sample with c-axis orientation. In our opinion, such a low value of J_c_ was mainly originated from the following two reasons. Firstly, the c-axis texture of the bulk sample in this work was not strong enough, and other diffraction peaks of Yb123, such as (103) and (110), were still detected. Secondly, considering that homogenous distribution of finer-sized Yb211 inclusions is critical for improving the J_c_, but in this work, agglomerations and large size of Yb211 particles were observed in local region, as shown in Fig. 9. Therefore, more work needs to be done in the future focusing on the growth of texture and distribution of Yb211 phase to further improve the critical current density.

## Discussion

As presented above, Yb123 powder and bulk samples were prepared by solid-state reaction. And Yb123 sample with a higher phase purity could be obtained by annealing at 927 °C or 937 °C. Additionally, increasing the annealing temperature was conducive to prepare the highly c-axis oriented sample. However, based on above XRD results and DSC curves, it could be known that the solid-state reactions during the whole heat treatment process were complex and involved with the synthesis and decomposition of Yb123 phase. Therefore, providing a more extensive discussion on this matter was imperative.

According to the XRD patterns in Fig. 2b, besides the raw material phases, including Yb_2_O_3_, BaCO_3_ and CuO, large amounts of Ba–Cu–O phases (BaCuO_2_ and Ba_2_Cu_3_O_6_) were also detected while annealing at 830 °C. Therefore, the above Eq. (3) and the following reaction were mainly occurred.

6 $$ {\text{2BaCO}}_{{3}} + {\text{ 3CuO }} + { 1}/{\text{2O}}_{{2}} = {\text{ Ba}}_{{2}} {\text{Cu}}_{{3}} {\text{O}}_{{6}} + {\text{ CO}}_{{2}} . $$

In addition, at closer observation, the (110) diffraction peak of Yb123 (2θ = 33.0°) was also observed in the XRD pattern of the sample annealed at 830 °C, and none of Yb211 phase was identified (see Fig. 2b). Furthermore, a comparative experiment based on the Yb211–BaCuO_2_–CuO raw material system was carried out at the same temperature, and the result showed that none of Yb123 phase could be detected in the case. Therefore, the Yb123 phase at this temperature was directly synthesized from Yb_2_O_3_, BaCO_3_ and CuO, and the reaction was expressed by Eq. (6):

7 $$ {\text{2Yb}}_{{2}} {\text{O}}_{{3}} + {\text{ 8BaCO}}_{{3}} + {\text{ 12CuO }} + \, \left( {{1} - {\text{2y}}} \right){\text{ O}}_{{2}} = {\text{ 4YbBa}}_{{2}} {\text{Cu}}_{{3}} {\text{O}}_{{{7} - {\text{y}}}} + {\text{ 8CO}}_{{2}} . $$

It should be noted that the above reaction was correlated to four reactants simultaneously, which increased the difficulty of the reaction. And similar reactions were also reported in other literatures^35,52^. Moreover, no liquid phase was generated at this temperature, and the inherently-slow kinetics of solid-state reactions affected the formation of Yb123. Therefore, only a small amount of Yb123 was observed at this temperature ultimately.

With the annealing temperature increasing, a large amount of Yb211 was formed and gradually transformed to principal phase, as shown in Fig. 2b. In this temperature range, according to previous reports^35^, the reaction involved with the formation of Yb211 was expressed by:

8 $$ {\text{Yb}}_{{2}} {\text{O}}_{{3}} + {\text{ BaCO}}_{{3}} + {\text{ CuO }} = {\text{ Yb}}_{{2}} {\text{BaCuO}}_{{5}} + {\text{ CO}}_{{2}} \uparrow . $$

As the annealing temperature increased to 920 °C, YbBa_2_Cu_3_O_7-y_ was transformed to principal phase. The main reaction occurred in this temperature range was:

9 $$ {\text{Yb}}_{{2}} {\text{BaCuO}}_{{5}} + {\text{ 3BaCuO}}_{{2}} + {\text{ 2CuO }} + \, \left( {{1}/{2} - {\text{y}}} \right){\text{ O}}_{{2}} = {\text{ 2YbBa}}_{{2}} {\text{Cu}}_{{3}} {\text{O}}_{{{7} - {\text{y}}}} . $$

However, when the annealing temperature was higher than 920 °C, a competitive reaction was occurred resulting in the fluctuation of the content of second phase. In specific, Yb123 was formed from the reaction between Yb211 and BaCuO_2_. At the same time, the decomposition melting of Yb123 would generate Yb211 and liquid Ba–Cu–O compounds. In the temperature range of 920 °C to 927 °C, the annealing process was mainly dominated by the reaction of synthesizing Yb123 from Yb211 and BaCuO_2_.Therefore, the phase purity of Yb123 got increased with increasing the annealing temperature. However, as the annealing temperature increased to 930 °C, the decomposition of Yb123 was enhanced, which resulted in an increase in content of Yb211 and Ba–Cu–O phase. It was worth mentioning that this decomposition temperature was very close to the first endothermic peak (935 °C) of Yb123, which further confirmed the occurrence of Eq. (4). Furthermore, when the annealing temperature was above 930 °C, a certain amount of liquid phase could be formed during the holding process, which could significantly promote the regrowth of Yb123 from the peritectic reaction (Yb_2_BaCuO_5_(s) + [3BaCuO_2_ + 2CuO](l) + (1/2 − y) O_2_(g) = 2YbBa_2_Cu_3_O_7−y_(s)) during the cooling process^42^. Therefore, the phase purity of Yb123 was gradually increased again when the annealing temperature was above 930 °C. However, the phase purity of Yb123 was significantly decreased when the annealing temperature reached 940 °C (as shown in Fig. 3), suggesting that when the annealing temperature was above 937 °C, the whole process was dominated by the decomposition of Yb123 phase, and the regrowth of Yb123 in the cooling process was insufficient. In fact, as presented above, c-axis texture could be formed in the sample annealed above 940 °C, and the peak intensity of Yb123 (110) got decreased accordingly. Therefore, it would no longer be appropriate to characterize the phase purity based on the ratio of peak intensity in the case. Based on the analysis, the main reactions involved in the experiment during heat treatment are presented in Table 2.

**Table 2:** Reactions occurred in flowing oxygen based on Yb_2_O_3_-BaCO_3_-CuO system at different annealing temperatures.

T (℃)	Main reaction
~ 830	BaCO_3_ + CuO = BaCuO_2_ + CO_2_↑
	2BaCO_3_ + 3CuO + 1/2O_2_ = Ba_2_Cu_3_O_6_ + CO_2_
	2Yb_2_O_3_ + 8BaCO_3_ + 12CuO + (1 − 2y) O_2_ = 4YbBa_2_Cu_3_O_7−y_ + 8CO_2_
> 850	Yb_2_O_3_ + BaCO_3_ + CuO = Yb_2_BaCuO_5_ + CO_2_↑
920–927	Yb_2_BaCuO_5_ + 3BaCuO_2_ + 2CuO + (1/2 − y) O_2_ = 2YbBa_2_Cu_3_O_7−y_
927–930, > 937	YbBa_2_Cu_3_O_7−y_ ↔ *a*Yb_2_BaCuO_5_ + *b*L + *c*O_2_↑
930–937	Yb_2_BaCuO_5_ + [3BaCuO_2_ + 2CuO] (l) + (1/2 − y) O_2_ = 2YbBa_2_Cu_3_O_7-y_

## Conclusions

In this work, Yb123 superconductor was prepared from Yb_2_O_3_, BaCO_3_ and CuO by solid-state reaction in flowing oxygen. The phase transformation process and the growth of texture for the bulk sample during annealing were systematically studied. The following conclusions could be drawn:

1. The formation mechanism of Yb123 during annealing could be divided into three different cases: at an annealing temperature below 850 °C, Yb123 was mainly synthesized from raw materials; as the annealing temperature increased to 937 °C, the formation of Yb123 was mainly originated from the solid-state reaction between Yb211, BaCuO_2_ and CuO; accordingly, at an annealing temperature higher than 937 °C, the formation of Yb123 was closely related to the regrowth process from liquid phase by the peritectic reaction during cooling.
2. The Yb123 sample with the highest phase purity could be obtained while annealing at 927 °C and 937 °C, respectively. And quantitative analysis showed that the phase purity of the sample annealed at 937 °C exceeded 80 wt%.
3. A relatively higher annealing temperature was beneficial for the growth of c-axis texture for Yb123 bulks. The bulk sample annealed at 937 °C exhibited a strong c-axis texture and its superconducting results indicated that the critical transition temperature was 89.9 K, and the self-field critical current densities at 4.2 K and 77 K were 1.3 × 10^5^ A/cm^2^ and 5.0 × 10^3^ A/cm^2^, respectively.

## Data Availability

The datasets used and/or analysed during the current study available from the corresponding author on reasonable request.
